# A Septin-Dependent Diffusion Barrier at Dendritic Spine Necks

**DOI:** 10.1371/journal.pone.0113916

**Published:** 2014-12-10

**Authors:** Helge Ewers, Tomoko Tada, Jennifer D. Petersen, Bence Racz, Morgan Sheng, Daniel Choquet

**Affiliations:** 1 Université de Bordeaux, Interdisciplinary Institute for Neuroscience, UMR 5297, F-33000 Bordeaux, France; 2 CNRS, Interdisciplinary Institute for Neuroscience, UMR 5297, F-33000 Bordeaux, France; 3 The Picower Institute for Learning and Memory, Departments of Brain and Cognitive Sciences and Biology, Massachusetts Institute of Technology, Cambridge, MA 02446, United States of America; 4 Department of Anatomy and Histology, Faculty of Veterinary Science, Szent István University, 1078 Budapest, Hungary; Georgia Regents University, United States of America

## Abstract

Excitatory glutamatergic synapses at dendritic spines exchange and modulate their receptor content via lateral membrane diffusion. Several studies have shown that the thin spine neck impedes the access of membrane and solute molecules to the spine head. However, it is unclear whether the spine neck geometry alone restricts access to dendritic spines or if a physical barrier to the diffusion of molecules exists. Here, we investigated whether a complex of septin cytoskeletal GTPases localized at the base of the spine neck regulates diffusion across the spine neck. We found that, during development, a marker of the septin complex, Septin7 (Sept7), becomes localized to the spine neck where it forms a stable structure underneath the plasma membrane. We show that diffusion of receptors and bulk membrane, but not cytoplasmic proteins, is slower in spines bearing Sept7 at their neck. Finally, when Sept7 expression was suppressed by RNA interference, membrane molecules explored larger membrane areas. Our findings indicate that Sept7 regulates membrane protein access to spines.

## Introduction

Diffusional exchange of molecules between the dendritic membrane and excitatory synapses is important for the dynamic, activity-dependent change in synapse composition that underlies some forms of synaptic plasticity. Diffusion also contributes to the turnover of desensitized receptors during synaptic transmission [1], [2] and the redistribution of components during the formation and breakdown of synapses [3]–[5]. Most excitatory synapses in mature neurons are located on small protrusions from the dendrite shaft termed spines. Typically the bulbous spine head bearing the postsynaptic density is separated from the dendrite shaft by an elongated neck. This neck is thought to impede the flow of solute and membrane molecules from the dendrite into the spine head and vice-versa. However, while neck width and length seem to play a role in modulating molecular exchange [3], [6], [7], the mechanism for this restriction is not clear.

Recently, a novel protein complex was described that localizes to dendritic branching points and spine necks and is required for dendritic arborization [8], [9]. This complex is comprised of at least three members of the septin family of GTPases, namely septin 5, septin 7 and septin 11 [9], and biochemically associates with membranes [8]. The crystal structure of a mammalian septin complex has been solved [10], in which hetero-hexameric complexes assemble end-over-end into filaments and rings. Such structures have been reported for the evolutionarily conserved septins in many systems, most prominently for budding yeast, where a septin ring surrounding the cleavage furrow forms a diffusion barrier between the two separating cells [11], [12]. Septin-dependent membrane partitioning seems to be ubiquitous in biology [13], raising the possibility that septins may form a diffusion barrier at the spine neck as well [14].

Here we investigated the localization, structure and stability of the spine-localized septin complex in cultured hippocampal neurons by fluorescence and electron microscopy and asked whether the presence of Sept7 at spine necks influences membrane protein mobility across the spine neck. In particular, we tested if the presence of septin at the spine neck alters the diffusion of AMPA-type glutamate receptors (AMPARs), the diffusion of which into and out of synapses regulates the strength of synaptic transmission and short-term synaptic plasticity [1]. We found that Sept7-GFP is part of a stable, membrane-aligned structure that becomes localized with high fidelity to dendritic spine necks during development. Single particle tracking showed that GluA2 subunit-containing AMPARs move slower and dwell longer in Sept7-positive spines. Furthermore, in fluorescence recovery after photobleaching (FRAP) experiments, transmembrane molecules and molecules attached to the inner membrane leaflet were restricted in their flow into Sept7-positive spines, while outer leaflet associated GPI-anchored molecules and soluble molecules were not detectably restricted. Finally, we found that RNAi-mediated reduction of Sept7 expression led to increased spine surface exploration of transmembrane proteins. We conclude that Sept7 at dendritic spine necks restricts membrane protein but not cytoplasmic flow across spine necks.

## Results

**Figure 1:**
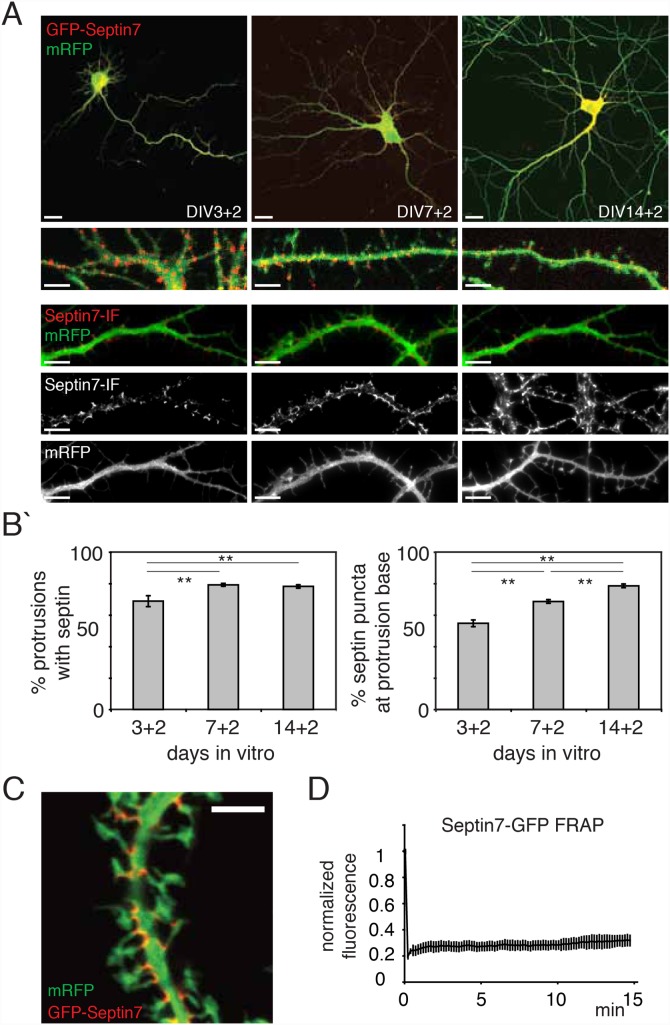
A membrane-aligned, stable Sept-positive structure locates to dendritic spine necks. (a) Fluorescence images of fixed primary hippocampal neurons at various days in vitro (DIV) expressing Sept7-GFP and mRFP for two days (+2). Scale bars are 20 µm. Below are magnified regions to clarify the localization of Sept7-GFP (first row) and immunostained endogenous Sept7 (second row) respectively, these are also shown as single channel mages at the bottom to emphasize the discrete localization of septins at the base of protrusions. Scale bars are 5 µm. (b) Quantification of experiments as shown in (a). Over time, more protrusions bear Sept7-GFP at their neck (left) and Sept7-GFP spots are more likely to be localized to protrusion necks (right). Histograms show mean ± SEM; **p<0.01, in One-way ANOVA. n>30 neurons in >3 independent experiments. (c) Confocal images of fixed hippocampal neurons at DIV 21 after 2 weeks of Sept7-GFP (pseudocolored red) and mRFP (pseudocolored green) expression. Sept7-GFP staining is concentrated in arc-like structures aligning spine necks. (d) Quantification of fluorescence recovery after photobleaching experiments on Sept7-GFP complexes in DIV 7 neurons. Shown is the average recovery measured of a total of 345 spotlike Sept7-GFP structures in 31 cells in 4 cultures with SEM.

Sept7-containing complexes localize to dendritic branching points and to the neck of dendritic filopodia and spines [8], [9] To further examine the generation of this pattern during the morphological development of neurons, we expressed Sept7-GFP in cultured neurons at different time points (Fig. 1a). Expression of Sept7-GFP resulted in a characteristic pattern of discrete spots that exhibited the same distribution as immunofluorescence staining of endogenous Sept7 (Fig. 1a, bottom row), suggesting that Sept7-GFP localizes and assembles into discrete structures that are indistinguishable from endogenous Sept7.

By quantifying the localization of Sept7-GFP positive spots during neuronal development, we found that with time, more and more of the protrusions emanating from the dendrites harbored Sept7-GFP staining at their necks. The percentage of Sept7-positive protrusions rose form 65% at 3+2 days in vitro (DIV) to 80% at 14+2 DIV (Fig. 1b). During the same period, Sept7-GFP became localized to protrusion necks with increasing fidelity until finally at DIV 14+2 more than 80% of all Sept7-GFP spots were localized to protrusion necks (Fig. 1b). These results suggest that the specific localization at the spine neck reflects an important aspect of Sept7 function.

For a detailed investigation of the Sept7 structure at mature spine necks, we let neurons express Sept7-GFP for 2–3 weeks and analyzed its localization by confocal microscopy. We found that spine neck Sept7-GFP formed arc-like structures along the plasma membrane (Fig. 1c). In neurons with very high Sept7-GFP expression, rings could occasionally be observed in the cytoplasm but not surrounding the base of spines and spine necks (data not shown). We concluded that Sept7 forms thin elongated complexes underlying the plasma membrane at the dendritic spine necks.

**Figure 2:**
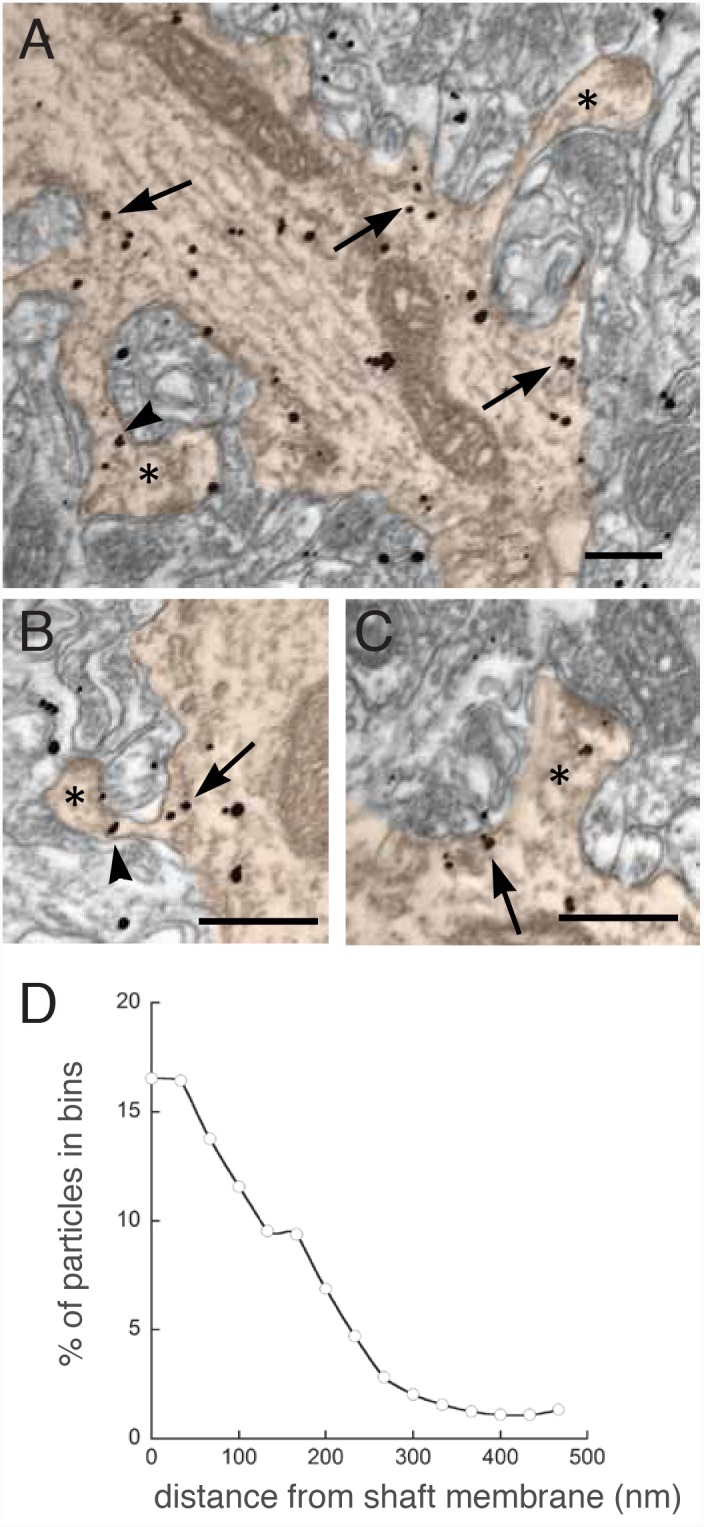
Immunoelectron microscopy of Sept7 in hippocampus. (a–c) Electronmicrographs showing the neuropil of CA1 stratum radiatum; spines (asterisks) and dendritic shafts are highlighted in orange false color. Particles coding for Sept-7 were often concentrated at the base of the spine (arrows) within the dendritic shaft. Labeling was seen not only in spine heads (a, c) but also in spine necks (arrowheads in a, b). Scale bars, 200 nm. (d) Line graph showing a quantification of particle localization in respect to the distance from the dendritic plasma membrane. Approximately 60% of the immunogold particles are concentrated within 0–100 nm from the membrane. The line graph was smoothened digitally using a three-point weighted running average.

To specify Sept7 distribution more precisely, we performed electron microscopy of Sept7 in rat hippocampus using pre-embedding immunogold labeling. We found numerous electron-dense particles in dendrites, spines and axon terminals. While particles were present in dendrites and spines, they typically concentrated at the base of the spine neck, where spines were attached to the dendritic shaft (Fig. 2a–c, Table 1). Axon terminals were also labeled, but not studied further. To establish the distribution of Sept7 within dendritic shafts, we measured the distances of gold particles from the shaft membrane. Finding no obvious differences, we pooled the samples from three animals for subsequent analysis. Consistent with our observation with fluorescence microscopy of Sept7-GFP and endogenous Sept7 (Fig. 1c), gold particles labeling Sept7 concentrated in the vicinity of the dendritic membrane, at a mean distance of 146±9 nm (n = 250 particles), with a peak labeling density of ∼50 nm from the membrane (Fig. 2d). Interestingly, gold particles labeling Sept7 were often associated with large (∼35–50 nm in diameter), elongated and electron dense structures at spine necks (Fig. 2b–c, arrows). In cultured hippocampal neurons, we found a similar distribution with prominent staining at the arc-shaped dendritic spine base (S1 Figure). Thus, immuno-EM shows that Sept7 is heavily concentrated directly underneath the dendritic membrane, and particles labeling Sept7 concentrate at the base of spines.

**Table 1:** Densities of gold particles coding for Sept7 in different subcellular compartments.

	Density (particles/µm^2^)
Nucleus	1.21±0.11 (n = 24)
Dendritic shaft	8.65±1.0 (n = 52)*
Spine	14.24±2.9 (n = 100)*
Axon terminal	16.24±3.6 (n = 100)*

Randomly selected profiles identifiable as axon terminals, dendritic shafts, or spines were included in the analysis, regardless of whether they were immunopositive. All data were collected from CA1 stratum radiatum, 4.0 mm caudal to bregma. To assess noise, nuclear profiles from the stratum pyramidale were also examined. Dendritic shafts, axon terminals and spines showed significant differences from the nuclear background. *p<0.001; two-sided t test (mean ± SE; n = number of profiles).

The septin ring at the yeast bud-neck oscillates between a fluid state with rapid exchange of septin molecules and a very stable state [15]. When we photobleached Sept7-GFP complexes at individual spine necks and then acquired time-lapse images for 15 minutes (S1 Movie), we found no recovery of Sept7-GFP fluorescence over the entire period (Fig. 1d, S1 Movie), independent of the stage of neuronal development. We conclude that there is no steady-state Sept7 turnover in septin complexes at this timescale.

**Figure 3:**
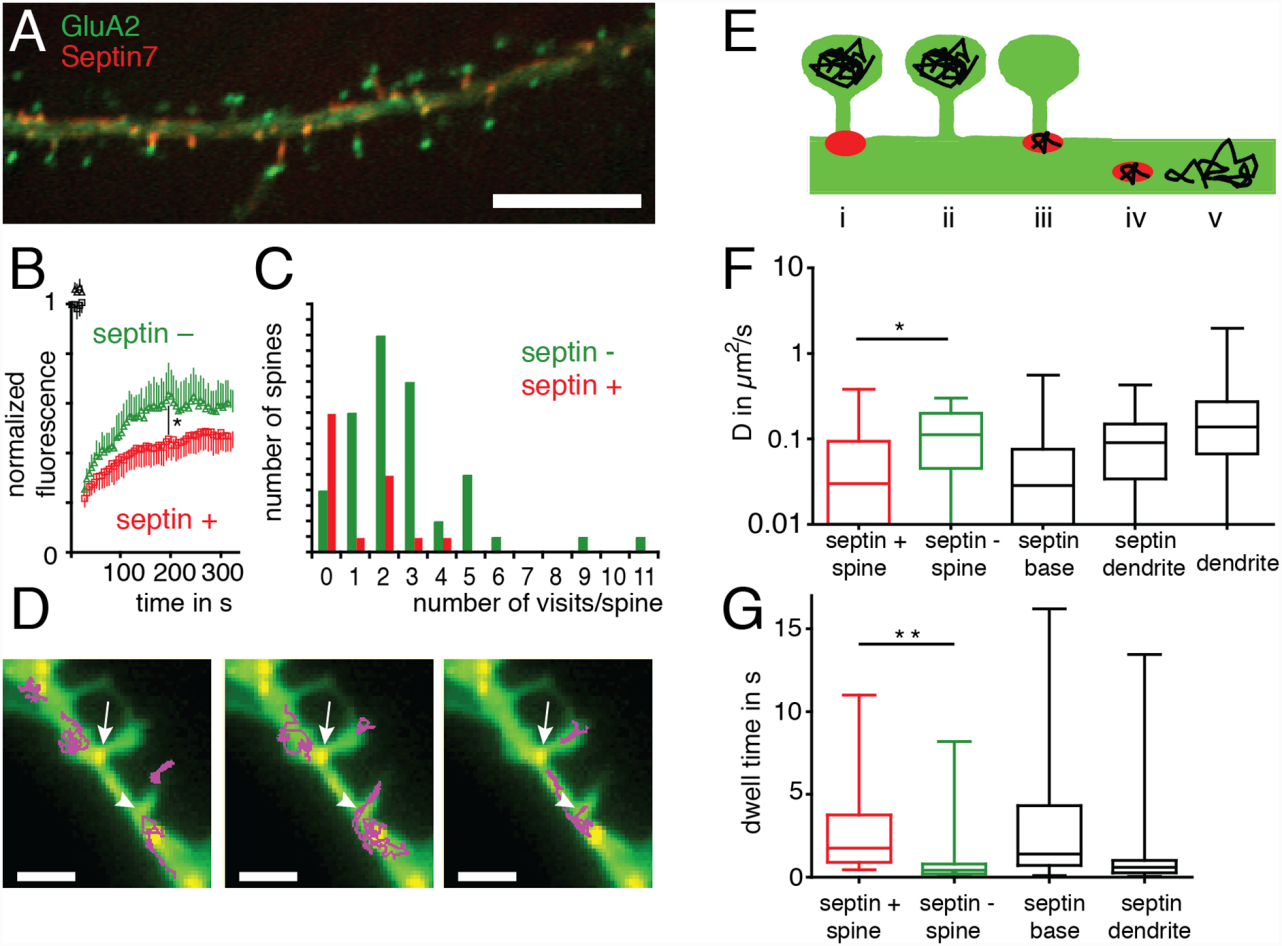
Membrane-dynamics of GluA2 in respect to Sept7 localization to spine necks. (a) Fluorescence microscopy image of a region of interest from a DIV 10+2 hippocampal neuron transiently expressing GluA2-SEP and Sept7-mOrange2. GluA2-SEP staining is enriched at the tip of dendritic spines, whereas Sept7-mOrange2 positive areas are found at the spine base. Scale bar is 10 µm. (b) Quantification of GluA2 fluorescence recovery in Sept7-GFP-positive (red squares) and Sept7-GFP-negative spines (green triangles). More than 10 spines from more than three independent experiments are utilized. (p = 0.0165) (c) Quantification of spine visits by GluA2-coupled quantum dots. An event is the crossing of the constricted area of the spine neck in the direction into the spine head area. Red bar: Sept7-GFP positive spines, green bar: Sept7-GFP negative spines. Shown is a statistic of all spines that bore mobile receptors in more than 30 movies from n>15 neurons. (d) Trajectories of single GluA2-coupled quantum dots from a single particle tracking experiment overlaid over a fluorescence image of the corresponding neuron. mRFP pseudocolored green, Sept7-GFP pseudocolored red, trajectories in magenta. Scale bar is 5 µm. (e) Classification of subcellular regions within dendrites for single particle tracking analysis from left to right: (i) the heads of spines that have Sept7-GFP staining at their necks. (ii) the heads of spines without Sept7-GFP at their neck. (iii) Sept7-GFP positive regions at spine bases, (iv) Sept7-GFP positive regions on the dendrite and (v) in Sept7-GFP free regions on the parent dendrite. (f) Analysis of GluA2-QD trajectories according to the diffusion coefficient. The median diffusion is reduced in Sept7-GFP positive spines compared with Sept7-GFP negative spines (bar: median±25–75% interquartile range, line: spread of data), *p<0.05, Mann-Whitney test. (g) Dwell time of GluA2 coupled QDs in different regions of the cell. The median dwell time is longer in Sept7-GFP positive spines (0.03 s) than in Sept7-GFP negative spines (0.11 s) (bar: median±25–75% interquartile range, line: spread of data), **p<0.01, Mann-Whitney test. Shown are experiment performed in 3 cultures in 32 cells with a total of 286 QDs.

A stable sub-membraneous cytoskeletal complex may sterically hinder the mobility of receptors in the plane of the plasma membrane by acting as a physical barrier. To investigate the dependence of receptor mobility on Sept7 presence at the spine neck, we expressed mOrange2-labelled Sept7 together with the GluA2 subunit of AMPARs coupled via its extracellular domain to superecliptic pHluorin (SEP), a pH-sensitive green fluorescent protein [16] (Fig. 3a). This labeling method ensures that only GluA2 subunits functionally inserted into the plasma membrane are fluorescent, while molecules in endosomal compartments that exhibit a lower pH are not detected. Hence the fluorescence-recovery in this experiment measures only plasma membrane-inserted AMPARs and not intracellular AMPAR pools. When we photobleached GluA2-SEP fluorescence in individual spines and monitored fluorescence recovery in time lapse imaging, we found that in Sept7-mOrange2 positive spines, fluorescence recovery was markedly slower than in Sept7-mOrange2 negative spines (Fig. 3b), suggesting that AMPAR diffusion into and/or out of spines is reduced by the presence of Sept7.

To further investigate this phenomenon, we performed single particle tracking (SPT) of endogenous GluA2-containing AMPARs labeled with semiconductor quantum dots (QDs) on Sept7-GFP and mRFP expressing neurons (S2 Movie). Using SPT, we tracked the diffusion of individual receptors that were in the plasma membrane of synaptic spines to measure the frequency at which they crossed spine necks. Every crossing of the spine neck was counted as an event. We found that AMPARs entered and exited Sept7-GFP positive spines less frequently than they crossed the necks of Sept7-GFP negative spines (Fig. 3c, d).

For a more detailed analysis, we segmented all individual QD trajectories according to their relative localization to five different regions of the cell and calculated the diffusion coefficient for each region: (i) Sept7-GFP positive spines, (ii) Sept7-GFP negative spines, (iii) Sept7-GFP areas at spine necks, (iv) Sept7-GFP areas elsewhere on the dendrite and (v) Sept7-GFP negative dendrite area (Fig. 3e). The diffusion coefficient of GluA2-coupled QDs was significantly lower in Sept7-GFP positive spines than in Sept7-GFP negative spines. QDs located to Sept7-GFP staining at the spine neck also exhibited low mobility (Fig. 3f). When we quantified the time each receptor spent in those regions (dwell times), we found that GluA2-QDs spent significantly more time in Sept7-GFP positive spines and Sept7-GFP-positive areas at the spine neck than in Sept7-GFP negative spines and dendritic Sept7-GFP-positive areas (Fig. 3g). The lower diffusion coefficient values and longer dwell times in the Sept7-GFP positive spines suggest that Sept7-GFP regions at the neck could represent a diffusion barrier between dendrite and spines.

**Figure 4:**
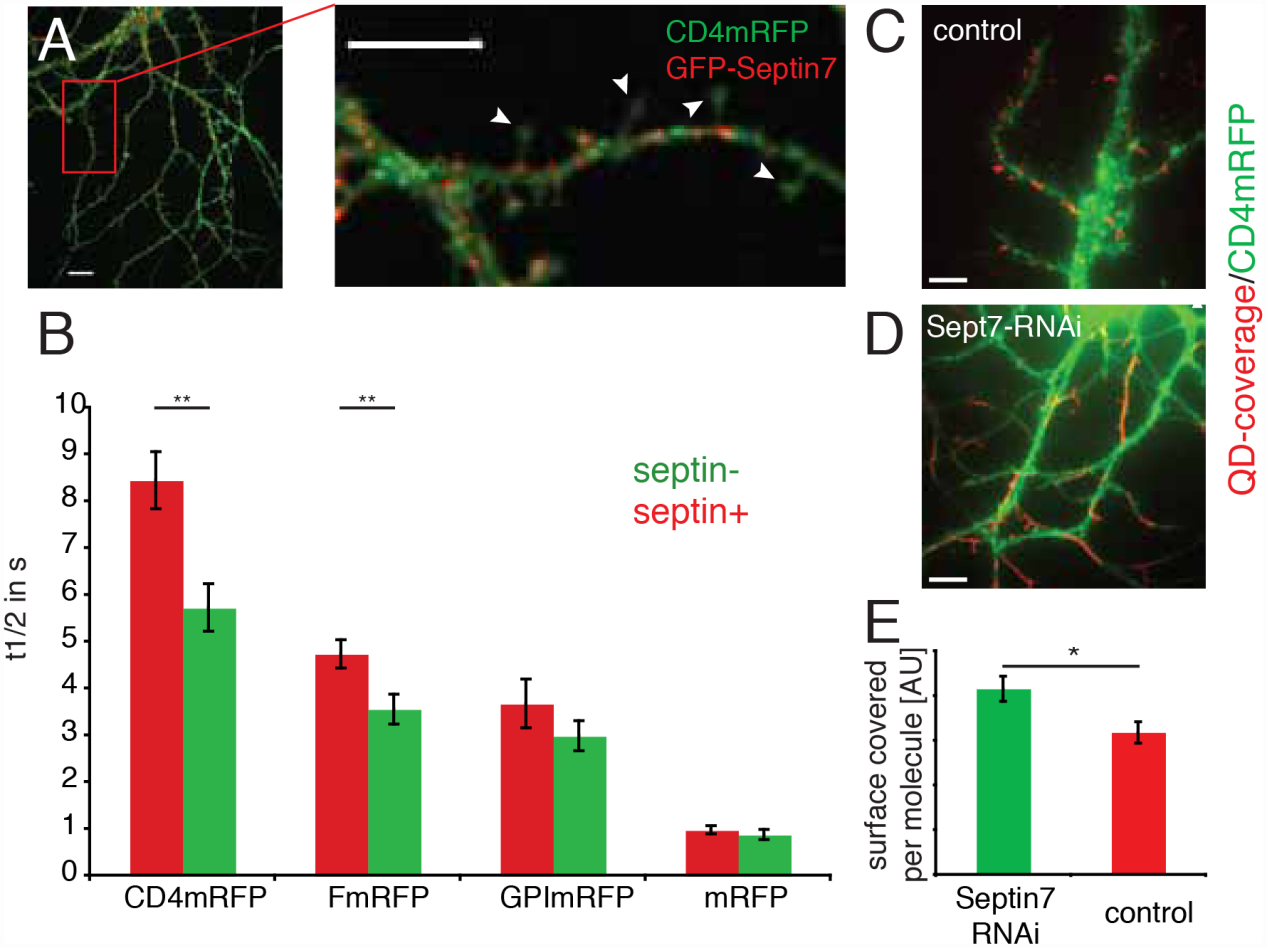
Sept7 and membrane flow in dendritic spines. (a) Fluorescence image of hippocampal neuron expressing Sept7-GFP and CD4-mRFP for 2 weeks (DIV7+14), inset is showing spines that were selected for FRAP analysis. Scale bar is 5 µm. (b) Graph of CD4-mRFP, F-mRFP, GPI-mRFP and mRFP fluorescence-recovery halftimes in Sept7-GFP positive (red) and Sept7-GFP negative (green) spines. All experiments were done in ≥3 neurons in ≥10 spines. **p<0.01, Mann-Whitney test. (c, d) Maximum projection of movies of CD4-mRFP coupled QDs on neurons transfected with control RNAi (c) or Sept7 RNAi (d), illustrating the total area browsed by diffusing molecules (red). The CD4-mRFP staining of the cell is shown in green. Note that on Sept7 RNAi treated neurons QDs move great distances and browse many spines. Scale bars are 5 µm. (e) Quantification of CD4-mRFP mobility on Sept7 (green) or control (red) shRNA treated cells. Shown is the absolute area covered per quantum dot in µm^2^. *p<0.05, Mann-Whitney test. Shown are experiment performed in 3 cultures in 31 cells with a total of 994 QDs.

GluA2-containing AMPARs can be immobilized at synapses by interacting with scaffold molecules [17]–[20], suggesting that the reduced mobility of AMPARs at the synapses could result from an increased number of AMPAR interactions with such scaffold molecules in the postsynaptic density at Sept7 positive spines, rather than a role of Sept7 as a diffusion barrier. To clarify whether membrane flow across the neck of Sept7 positive spines is indeed reduced, we used FRAP to measure the diffusion properties in spines of 4 different mRFP-tagged molecules: CD4-mRFP (a single transmembrane domain spanning protein), F-mRFP (an inner membrane leaflet marker), GPI-mRFP (an outer membrane leaflet marker) and mRFP (a soluble cytoplasmic marker) (Fig. 4 and S2b Figure). Each protein was co-expressed with Sept7-GFP for 10 to 20 days and exhibited membrane fluorescence with no apparent enrichment in dendritic spines (Fig. 4a, S2 Figure), demonstrating a homogeneous distribution in the plasma membrane. When the CD4-mRFP fluorescence in single spines was photobleached by a short pulse of a 488 nm laser line, the fluorescence recovered within seconds almost completely (S3 Movie). In fact, consecutive experiments on the same spine were highly reproducible and could be fit by a single exponential, verifying that only membrane flow was responsible for fluorescence recovery (S3 Figure). To avoid a possible influence of spine shape on membrane flow across the neck of dendritic spines, spines having similar length and neck width were selected for experiments (S4 Figure).

We found that CD4-mRFP displayed a slower recovery rate in Sept7-GFP positive than Sept7-GFP negative spines (Fig. 4b and Table 2; p = 0.0019).

**Table 2:** Recovery times of fluorescent molecules in spines.

Molecule	Sept7-GFP +	Sept7-GFP –	p
CD4-mRFP	8.44±0.61 s (n = 29)	5.72±0.51 s (n = 34)	0.0019**
F-mRFP	4.73±0.30 s (n = 16)	3.55±0.32 s (n = 12)	0.0088**
GPI-mRFP	3.67±0.52 s (n = 18)	2.98±0.32 s (n = 20)	0.4058 n.s.
mRFP	0.97±0.09 s (n = 15)	0.87±0.11 s (n = 10)	0.5823 n.s.

Comparison of the recovery-halftimes in FRAP of transmembrane (Cd4-mRFP), inner membrane leaflet (F-mRFP), outer membrane leaflet (GPI-mRFP) and soluble (mRFP) fluorescent proteins. Compared are average values for spines with Sept7-GFP at their bases (Sept7-GFP +) with values for spines that do not bear detectable Sept7-GFP staining at their bases. Shown are the absolute recovery halftimes from single exponential fits to at least three averaged recovery curves per spine. The numbers show mean ± SEM; **p<0.01, Mann-Whitney test.

Since Sept7-GFP seems to be aligned with the inner membrane leaflet, we wondered whether the specific localization in respect to the plasma membrane would influence fluorescence recovery in dendritic spines. To investigate this, we targeted mRFP by a farnesyl anchor to the inner membrane leaflet (F-mRFP) or by a GPI-anchor to the extracellular membrane leaflet (GPI-mRFP) or expressed soluble mRFP (mRFP). We found that the flow of F-mRFP (p = 0.0088), but not that of GPI-mRFP and mRFP, was reduced in Sept7-GFP positive spines (Fig. 4b, Table 2). Taken together, these results suggest that the diffusion of molecules spanning the plasma membrane and molecules anchored to the inner membrane leaflet are reduced by the presence of Sept7 at the spine neck, but cytoplasmic proteins or molecules anchored to the outer membrane leaflet are not impeded in their diffusion.

To investigate whether Sept7 was required to reduce access to spine heads, we knocked down its expression using shRNA, as we have established and quantified before [8]. Using SPT, we tracked the diffusion of quantum dots bound to CD4-mRFP in neurons co-transfected with CD4-mRFP as a marker and either shRNA directed against Sept7 or a control shRNA plasmid against GFP. (Fig. 4c and d). We found that in Sept7-shRNA-expressing neurons, QDs frequently crossed spine necks and explored many protrusions. On the other hand, in control neurons, mobile QDs were often confined to spines or small areas on the dendrite. When we quantified the total area covered by quantum dots, we found that the area covered by CD4-mRFP coupled QDs was larger in neurons treated with Sept7 shRNA than in control neurons (Fig. 4e).

## Discussion

Sept7 complexes became localized to the base of dendritic spine with high efficiency and fidelity during filopodial outgrowth and spine development. At the base of dendritic spines, Sept7-GFP formed arc shaped structures that aligned with the plasma membrane. This finding agrees with previous data on mammalian septins. Septins are known to bind to acidic phospholipids *in* *vitro* [21], and membrane-binding and filament formation of brain-derived septin complexes can lead to induction of negative curvature on liposomes [22]. Interestingly, this membrane bending activity was strongest when septins bound to PIP2 in the liposomes, a lipid that is enriched in dendritic spines [23]. Both the presence of PIP2 and the preference for negatively curved membranes may be responsible for the recruitment of Sept7 to dendritic spine necks. Confocal imaging of GFP-tagged Sept7 showed thin, elongated, arc-like Sept7-containing structures aligning the plasma membrane at spine necks consistent with reported filament formation of mammalian septins [9], [10], [24], [25]. We could not detect septin rings at dendritic spine necks by fluorescence or electron microscopy, although gold particles were localized consistently in the vicinity of large electron dense structures that resemble the Sept7 complex. The septin structure at the yeast bud-neck seems to be rather fluid when it is first assembled, however once the ring encircles the bud, there is virtually no turnover except for a short phase of structural reorganization of the complex [15]. For the spine neck septin complex, we did not detect major structural rearrangements or significant Sept7-GFP turnover during 15 minute FRAP experiments, suggesting a similarly stable structure. More detailed studies of septin enrichment, organization and turnover are required to elucidate the dynamics and possible filamentous nature of the spine neck septin complexes.

Using SPT, we observed that QD-coupled AMPARs moved slowly and dwelled for long times in Sept7-GFP-positive spines and on Sept7-GFP regions at the spine neck. The Sept7-GFP positive regions at the spine neck could reduce GluA2 diffusion by increasing local viscosity. However, the septin diffusion barrier we report here is different from the diffusion barriers identified at the yeast bud-neck [11], [12], the cilial base [26] or the initial segment of the axon [27], [28] which abolish the diffusion of proteins and lipids across them. The septin-dependent diffusion barrier we identified at the spine neck does not impart a complete block of membrane flow across the spine neck, but reduces it. Although we so far found no evidence for a continuous ring of septins surrounding the entire spine neck, it has recently been shown that septins form part of the cortical actin meshwork in heterologous cells [29] which is known to temporarily confine individual diffusing molecules in corrals [30]. Such a mechanism emphasized by undetectable differences in spine structure between Sept7-positive and Sept7-negative spines may reduce the flow of membrane proteins.

This would be consistent with our finding that only the diffusion of membrane bound, but not cytoplasmic molecules were restricted at septin-positive spines. It has been reported that long-term potentiation (LTP) stimulation to single spines lowered the threshold for LTP in neighboring spines [31], and this correlates with diffusion of membrane anchored, activated ras from the induced spine to the dendrite shaft [32]. This may be due to an incomplete circumference of the spine neck by the septin complex or to a less tight arrangement of the septin filaments at the plasma membrane. It will be interesting to study how synaptic stimulation alters the septin complex at the base of spines. The suggestion that submembranous Sept7 localization at the spine base allows it to act as a membrane diffusion barrier is supported by the observation that only the mobility of the transmembrane proteins (GluA2-SEP and CD4mRFP) or proteins linked to the intracellular leaflet (FmRFP) were reduced by septins.

The lateral diffusion of locally delivered AMPARs into and out-of synapses is a main route for constitutive exchange [3] and long-term potentiation of synaptic AMPARs [4], likely by recruitment of receptors to postsynaptic “binding slots” [18], [33]. Taken together with the finding that quick replacement of inactive receptors at the postsynapse by lateral diffusion is important for the signal recovery in paired pulses [2] and that the diffusion of solute molecules out of spine heads is activity-dependent [34], suggests that activity-regulated confinement of a reservoir of mobile receptors to the spine membrane by a septin mediated diffusion barrier might facilitate fast response to synaptic activity. Future work is aimed at clarifying a possible interdependence between Sept7 and synaptic plasticity.

## Experimental Procedures

### Cell culture and transfection

The care and use of animals was approved by the Experimental Ethical Committee of Bordeaux, (also called Comité d’éthique N°50 de Bordeaux). Cultures of hippocampal neurons were prepared from E18 Sprague-Dawley rats following a previously described method [35]. Time-mated animals were provided by a commercial provider and sacrificed by cervical dislocation after anesthesia. Cells were plated at a density of 100–200×10^3^ cells/ml on polylysine pre-coated coverslips and kept in serum free Neurobasal medium (Invitrogen) at 37°C in 5% CO2 for 16–18 days in vitro (DIV). During this period half of the medium was exchanged weekly. Neurons were transfected with a plasmid encoding for SEPT7-GFP [8] using Effectene (Qiagen) following the company protocol. A plasmid encoding soluble GFP, pEGFP-C1 (Clontech) was introduced using an Amaxa Nucleofector device (Lonza) following the manufacturer’s suggested protocol. All live cell microscopy was performed in extracellular solution (ECS, 145 mM NaCl, 5 mM KCl, 2 mM CaCl_2_, 1 mM MgCl_2_, 10 mM HEPES, 10 mM Glucose) supplemented with 0.2% bovine serum albumin at pH 7.4 and osmolarity-adjusted.

### Fluorescence recovery after photobleaching

Fluorescence recovery after photobleaching (FRAP) experiments were performed on a custom built spinning disk (Yokogawa) system with a separate laser bench for photobleaching (Roper Scientific) based on an automated Leica inverted microscope and equipped with a temperature-stable incubation chamber set to 37°C. Images were acquired using a QuantEM512SC electron multiplying charge-coupled device camera (Roper Scientific). Photobleaching experiments were performed using the multi-dimensional image acquisition routine in Metamorph (Molecular Devices). For fast FRAP experiments of membrane protein diffusion, up to 720 images were acquired in stream mode and spines were photobleached after the initial 20 frames of the stream acquisition. Up to 7 spines within a region of interest were photobleached with single 5 ms pulses of the 488 nm laser line. Image analysis, curve fitting and data analysis were performed in Image J, Prism and Excel. For statistical analysis, Mann-Whitney test was performed.

### Immunofluorescence staining of endogenous Septin7 in cultured neurons

At 5, 9 and 16 days in vitro (DIV), neurons expressing soluble GFP were fixed with 4**%** paraformaldehyde and 4**%** sucrose in PBS at room temperature for 10 min, and then rinsed 3 times in PBS before permeabilization with 0.1**%** Tx100 in PBS for 5 min. After 3 rinses in PBS, neurons were blocked for 60 min in blocking solution containing 1% BSA and 0.1% cold water fish skin gelatin (Sigma G7765) in PBS for 60 min. Next, neurons were incubated in rabbit anti septin7 primary antibody (IBL International GmbH, JP18991) diluted 1∶500 in blocking solution for 60 min, then incubated again in blocking solution before labeling with secondary antibody conjugated with Alexa 568 (Invitrogen) diluted in blocking solution (1∶1000) for 30 minutes. Coverslips were then rinsed several times in PBS, dipped in dH2O, and mounted in Mowiol mounting medium. Neurons were viewed using an epifluorescence microscope (Leica DM5000) using a 63x**/**1.4 NA objective. Images were acquired on a cooled CCD camera (CoolSNAP HQ2, Photometrix) and processed using MetaMorph imaging software.

### Single particle tracking

QD staining of surface AMPARs was performed as previously described [17]. Briefly, neurons were incubated with anti-GluA2 or anti-DsRed antibody for 10 min at 37°C, and then were incubated with QDs at a final concentration of 0.1 nM for 2 min at 37°C. For QD imaging, 1000 consecutive frames were acquired at 20 Hz with an EM-CCD camera (QuantEM, Roper Scientific) using Metamorph software (7.0, Universal Imaging Corp.). For EGFP and mRFP, images were obtained with an integration time of 50–100 ms. Samples were illuminated with a mercury lamp (Olympus, France). EGFP, mRFP and QD fluorescence signals were detected with appropriate excitation and emission filters controlled by automated filter wheels. Live cells were mounted onto an inverted microscope (IX71, Olympus, France) equipped with a 100X oil-immersion objective (NA = 1.4) for imaging resulting in a pixel size of 160 nm. Continuous tracking of single QDs was performed between blinks with custom software written within MATLAB (The Mathworks Inc., Natick, MA). The method is based on a QD maximal allowable displacement (4 pixels) during a maximal allowable duration of the dark period (25 frames, corresponding to 1.25 s acquisition). Instantaneous diffusion coefficients, D, were calculated as previously described [36] from linear fits of the n = 1 to 8 values of the mean squared displacement (MSD) *versus* time plot, according to the equation: MDS(t) = <r2> = 4Dt for 2D-diffusion. MSD(t) was calculated according to the formula: <r2> = [_i = 1_Σ_(N-n)_(X_i + n_–Xi)_2_+)(Y_i+n_–Yi)_2_/N–n]dt for reconstructed trajectories of more than 100 frames.

### Confocal microscopy

Confocal imaging was performed at the Bordeaux Imaging Center at University of BordeauxII using a Leica TCS SP2 laser scanning confocal microscope with excitation lines from a 488 nm and 543 nm lasers in the sequential mode. Samples for confocal microscopy were fixed in 4% paraformaldehyde with 4% sucrose in PBS and mounted in Vectashield (Vectorlabs, USA).

### Immunogold Electron Microscopy of Cultured Neurons

All reagents were purchased from Electron Microscopy Sciences unless otherwise stated and all steps carried out at room temperature. Coverslips with cultured hippocampal neurons (DIV19) were fixed with 4% paraformaldehyde in 0.15 M Sorensen’s phosphate buffer, pH 7.4 (PB) for 45 minutes. Next, cells were rinsed 2 times with PB and 2 times with Millonig’s phosphate buffered saline, 0.1 M, pH 7.4 (PBS). Cells were blocked and permeabilized for 60 minutes with PBS containing 1% bovine serum albumin (Sigma), 0.1% cold water fish skin gelatin, and 0.1% saponin (Sigma) (blocking solution). Primary antibody was diluted in the blocking solution and coverslips incubated on droplets of diluted antibody for 1.5 hours. Next cells were rinsed once in blocking solution and blocked again for 60 minutes. Cells were then incubated on droplets of FluoroNanogold anti mouse Fab’ Alexa Fluor 488 (Nanoprobes) diluted 1∶100 in blocking solution for 2 hours. Sometimes, at this stage the quality of FluorNanogold labeling was confirmed by epifluorescence microscopy before proceeding with electron microscopy. To proceed with electron microscopy, cells were fixed in freshly prepared 2% glutaraldehyde in PB for 30 minutes after the nanogold labeling. After rinsing in PB and water, the nanogold was silver intensified for 6–7 minutes using HQ Silver (Nanoprobes) according to manufacturer’s instructions, then rinsed thoroughly with ddH2O and returned to PB. Immediately after silver intensification, cells were post-fixed with 0.2% osmium in PB for 30 minutes. Then, cells were rinsed exhaustively with ddH2O before *en bloc* staining with 0.25% uranyl acetate in ddH2O for 30 minutes. After 3 water rinses, cells were dehydrated with rinses (3 minutes each) in a graded alcohol series (50%, 70%, 95%, 100%). Cells were infiltrated with EMbed812 starting at 70% in ethanol for 60 minutes, then two exchanges of 100% resin for at least 60 minutes each. Finally, coverslips were embedded by placing them cell side-up on a glass slide and then inverting resin-filled gelatin capsules on top of the coverslips. Coverslips were placed in a 60°C oven for 24–48 hours to allow polymerization. Polymerized samples were separated from coverslips and glass slides by gently warming over a flame until gelatin capsules popped free from glass. Samples were thin sectioned (60 nm sections; Leica EM-UCT) and picked up on formvar-coated copper mesh grids for viewing using a Hitachi 7650 transmission electron microscope operated at 80 kV. Images were captured using a Gatan Orius SC1000 CCD camera.

### Preparation of hippocampal tissue for electron microscopy

Experiments were carried out on 3–5 month old male Wistar rats from Toxi-COOP (Budapest, Hungary). Housing and experimental procedures were strictly in compliance with IACUC guidelines. Animals were deeply anesthetized with pentobarbital (60 mg/kg, i.p.), then perfused intracardially with saline, followed by a mixture of depolymerized paraformaldehyde (PFA; 4%) and glutaraldehyde (0.2%) in 0.1 M phosphate buffer pH 7.4; 50 µm coronal sections were cut with a Vibratome, and processed for immunohistochemistry.

### Immunocytochemistry of hippocampal tissue for electron microscopy

For immunogold labeling, floating sections were treated for 30 min in 1% sodium borohydride in PB, to quench free aldehyde groups. The sections were incubated in 20% normal donkey serum (NDS) for 20 min to suppress non-specific binding, and then for 12 h in anti-Human Sept7 antibody (1∶300, IBL International GmbH, JP18991), along with 2% NDS. Sections were incubated in biotinylated anti-rabbit IgG (Jackson, USA) for 2 h. Following rinses in PBS, sections were incubated in streptavidin coupled to 1.4 nm gold particles (1∶100, Nanoprobes Inc.) for 2 h at room temperature, and rinsed in PBS. Sections were washed in 0.01 M sodium acetate (to remove phosphate and chloride ions), followed by silver enhancement with IntenSE M kit (Amersham Biosciences).

Sections for electron microscopy were postfixed in 0.5–1% osmium tetroxide in 0.1 M PBS for 35–45 min and stained en bloc with 1% uranyl acetate for 1 h. After dehydration in ascending ethanol series and propylene oxide, sections were infiltrated with Durcupan resin (Fluka) and flat-mounted between sheets of Aclar (EMS) within glass slides. Seventy nm sections were cut, mounted on 300 mesh copper grids, contrasted with Sato’s lead, and examined in a JEOL T1100 electron microscope at 80 KV; images were collected with a 12 bit 1024×1024 CCD camera.

### Quantitative analysis of immunogold labeling in hippocampal tissue

Electron micrographs of randomly selected fields were taken from the proximal and middle regions of CA1 stratum radiatum. Synaptic profile areas and distances of gold particles from the membrane were measured using ImageJ v1.29 software (NIH, USA), see http://rsb.info.nih.gov/ij. For detailed description see refs: Racz and Weinberg (2004, 2006, 2008). To determine the relative density of Sept7 Both labeled and unlabeled profiles were included in the analysis. To assess the distribution of Sept7 in distinct subcellular compartments, we quantified the density of immunogold labeling: axon terminals, dendritic shafts, spines, and pyramidal cell nuclei were identified; gold particles within their cytoplasm were counted, and the areas were measured. Nonspecific background labeling was calculated by measuring particle-densities over the nuclei of pyramidal cells, because these were consistently immuno-negative in light microcopy and immuno-peroxidase or immunofluorescence staining (not shown). Dendritic shafts (∼8 times above background), terminals and spines (∼15 times above background) contained significantly higher concentrations of gold particles than did nuclei (p<0.001) (Table 2). Thus, Sept7 concentrates heavily in spines and terminals and dendritic shafts also contain Sept7 in hippocampal neurons. Particle densities for axonal, dendritic, and spine cytoplasm were computed and compared with nonspecific labeling, using a two-sided t test. Microsoft Excel and Kaleidagraph (Synergy Software, Reading, PA) were used to generate graphs and to compute statistics.

## Supporting Information

S1 Figure **Immunogold electron microscopy of endogenous Sept7 in cultured hippocampal neurons.** Electron micrographs showing spines of DIV19 cultured hippocampal neurons labeled for Sept7 with silver intensified immunogold. Labeling for Sept7 is concentrated at the plasma membrane of spine necks. Scale bars are 200 nm.
(TIF)

S2 Figure **Localization of 4 different mRFP tagged molecules in HEK cells and neurons.** (a) Detection of mRFP moiety of mRFP membrane constructs by antibody staining in HEK cells. Top row: merged images of cotransfected GFP with different mRFP constructs showing membrane localization for CD4-mRFP, F-mRFP and GPI-mRFP. The intracellular mRFP colocalizes with GFP. Second row: mRFP fluorescence alone. Third row: detection of mRFP by anti-mRFP surface immunostaining in un-permeabilized cells. The extracellular epitopes of CD4-mRFP and GPI-mRFP are accessible to antibody and stained. Fourth row: detection of mRFP by anti-DsRed surface immunostaining in unpermeabilized cells after cleavage of the GPI anchor by Phospholipase C. The mRFP moiety of GPI-mRFP is not detectable anymore. (b) Details of dendrites of neurons transfected with the same constructs.
(TIF)

S3 Figure **Fluorescence recovery after photobleaching experiments in individual spines.** (a) Raw fluorescence recovery data from eight consecutive measurements on the same spine. Note nearly complete recovery and roughly identical shape of recovery curves. (b) Normalization of the eight measurements in (a) shows identical recovery halftimes for all measurements.
(TIF)

S4 Figure **Shape of spines selected for FRAP experiments.** To rule out the influence of spatial constraints on the outcome of FRAP experiments, spines of similar apparent shape were selected for experiments. (a) Inverted fluorescence image of CD4-mRFP fluorescence in a dendrite. Selected spines are marked. Scale bar is 5 µm. (b) The average apparent neck width in µm of selected spines in septin positive and septin negative spines as measured from full width at half maximum of spine neck fluorescence profile. p = 0.3088. (c) Average spine length in µm of selected spines in septin-positive vs. septin negative spines. p = 0.5919. Mann-Whitney test.
(TIF)

S1 Movie **FRAP experiment on Sept7-GFP at the base of a spine.** The Sept7-GFP staining at the right spine neck is photobleached. For comparison, the Sept7-GFP staining at the left spine neck is not photobleached. Total time: 12 min.
(MOV)

S2 Movie **Single particle tracking of quantum dots coupled to GluA2 in DIV9 neurons expressing soluble mRFP (red) and Sept7-GFP (green).** The trajectories of several GluA2 coupled quantum dots are shown in cyan. While only few particles enter or exit the Sept7-GFP positive spine in the center of the image, the Sept7-GFP negative spine at the bottom is frequently entered and exited by GluA2 coupled quantum dots. Played in real time. Scale bar is 5 µm.
(MOV)

S3 Movie FRAP experiment of CD4-mRFP in a single Sept7-GFP positive spine. CD4-mRFP fluorescence is bleached in the spine by a pulse of 488 nm laser light and thereafter imaged by spinning disk confocal microscopy. Images are taken at 50 ms intervals. Played in real time.
(MOV)
