# Supplementary figures and images for: A Septin-Dependent Diffusion Barrier at Dendritic Spine Necks
Source: PLoS One. 2014 Dec 10;9(12):e113916. doi: 10.1371/journal.pone.0113916 (PMC4262254; doi:10.1371/journal.pone.0113916)

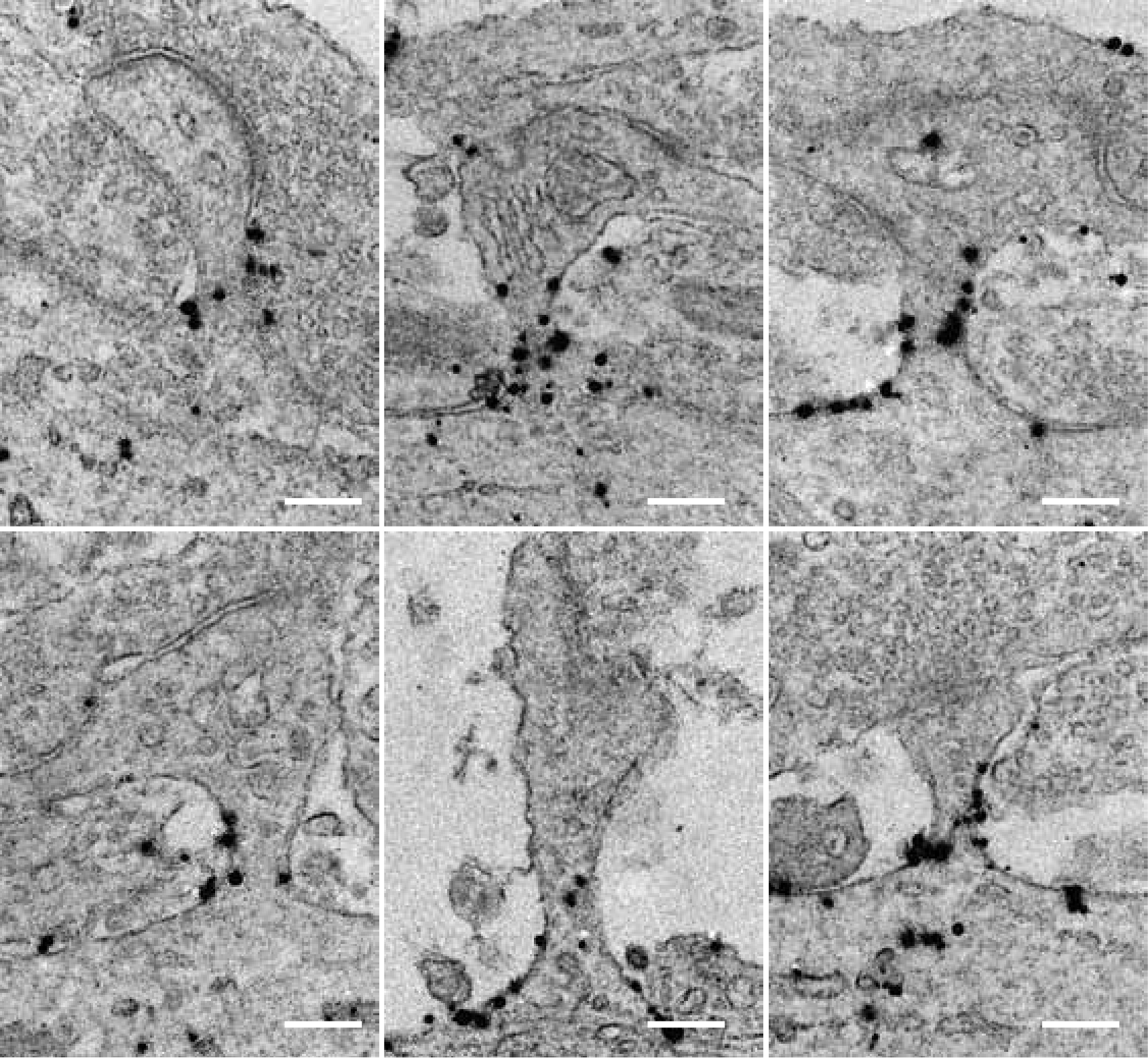

Supplement: S1 Figure — Immunogold electron microscopy of endogenous Sept7 in cultured hippocampal neurons. Electron micrographs showing spines of DIV19 cultured hippocampal neurons labeled for Sept7 with silver intensified immunogold. Labeling for Sept7 is concentrated at the plasma membrane of spine necks. Scale bars are 200 nm. (TIF) [file pone.0113916.s001.tif]

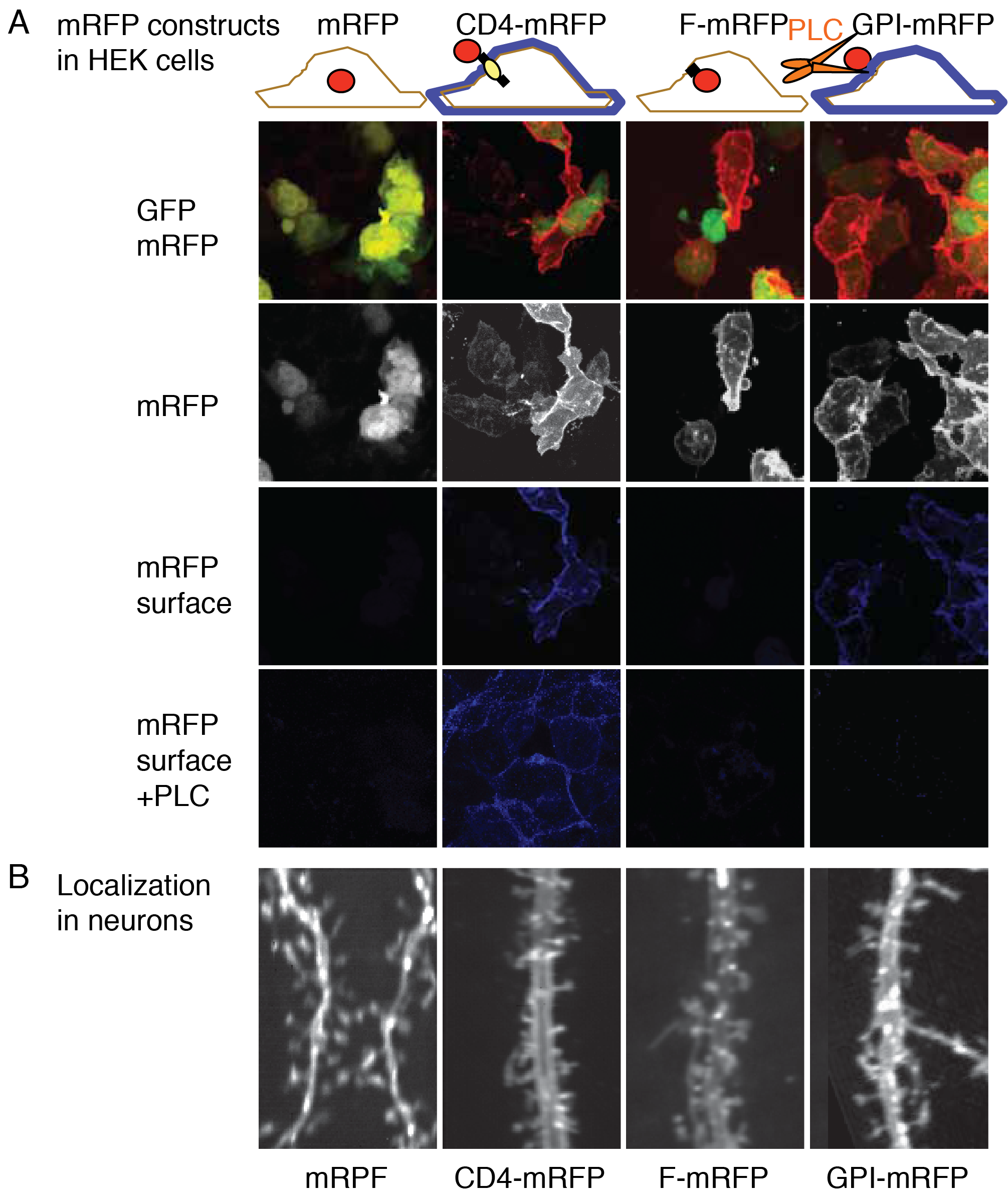

Supplement: S2 Figure — Localization of 4 different mRFP tagged molecules in HEK cells and neurons. (a) Detection of mRFP moiety of mRFP membrane constructs by antibody staining in HEK cells. Top row: merged images of cotransfected GFP with different mRFP constructs showing membrane localization for CD4-mRFP, F-mRFP and GPI-mRFP. The intracellular mRFP colocalizes with GFP. Second row: mRFP fluorescence alone. Third row: detection of mRFP by anti-mRFP surface immunostaining in un-permeabilized cells. The extracellular epitopes of CD4-mRFP and GPI-mRFP are accessible to antibody and stained. Fourth row: detection of mRFP by anti-DsRed surface immunostaining in unpermeabilized cells after cleavage of the GPI anchor by Phospholipase C. The mRFP moiety of GPI-mRFP is not detectable anymore. (b) Details of dendrites of neurons transfected with the same constructs. (TIF) [file pone.0113916.s002.tif]

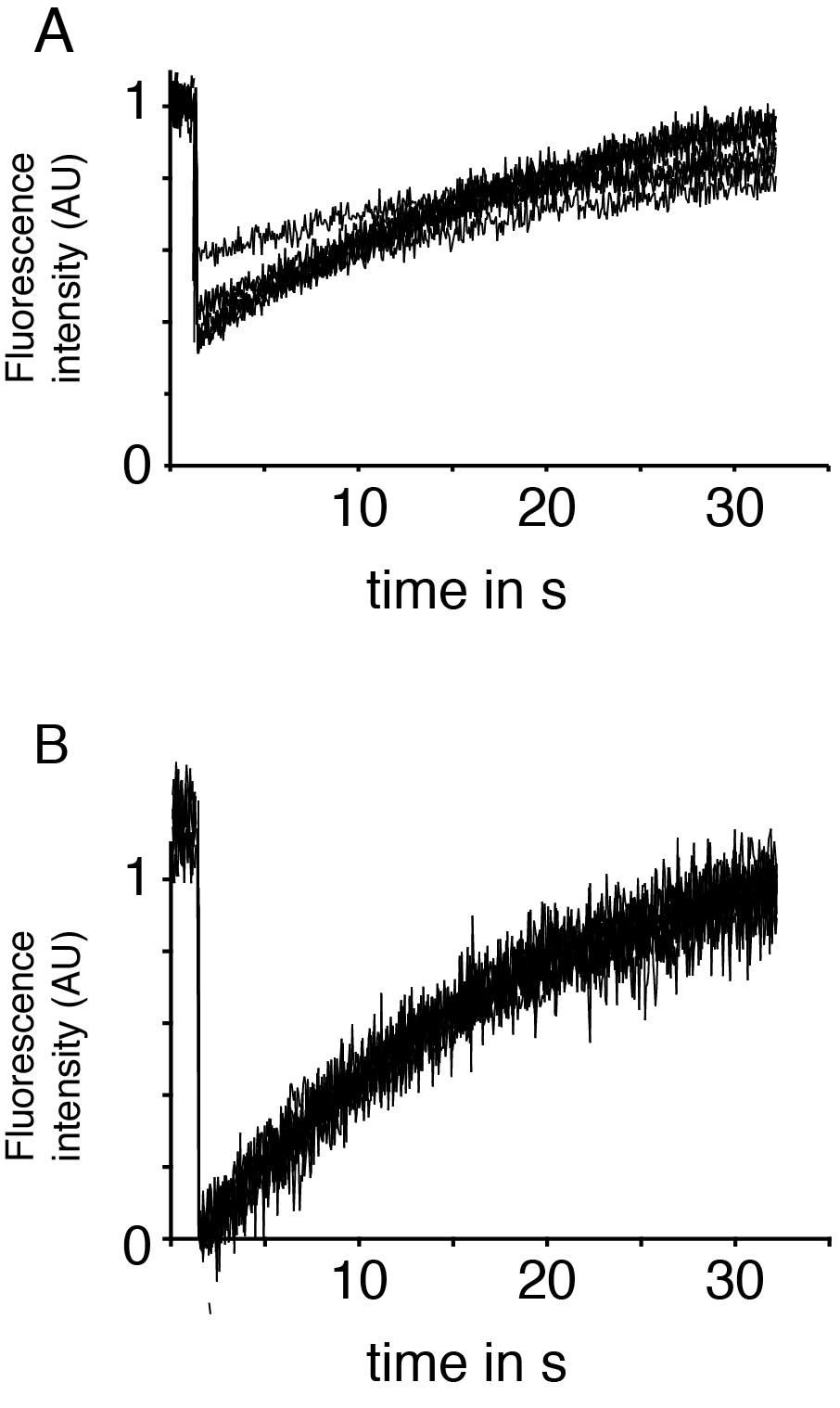

Supplement: S3 Figure — Fluorescence recovery after photobleaching experiments in individual spines. (a) Raw fluorescence recovery data from eight consecutive measurements on the same spine. Note nearly complete recovery and roughly identical shape of recovery curves. (b) Normalization of the eight measurements in (a) shows identical recovery halftimes for all measurements. (TIF) [file pone.0113916.s003.tif]

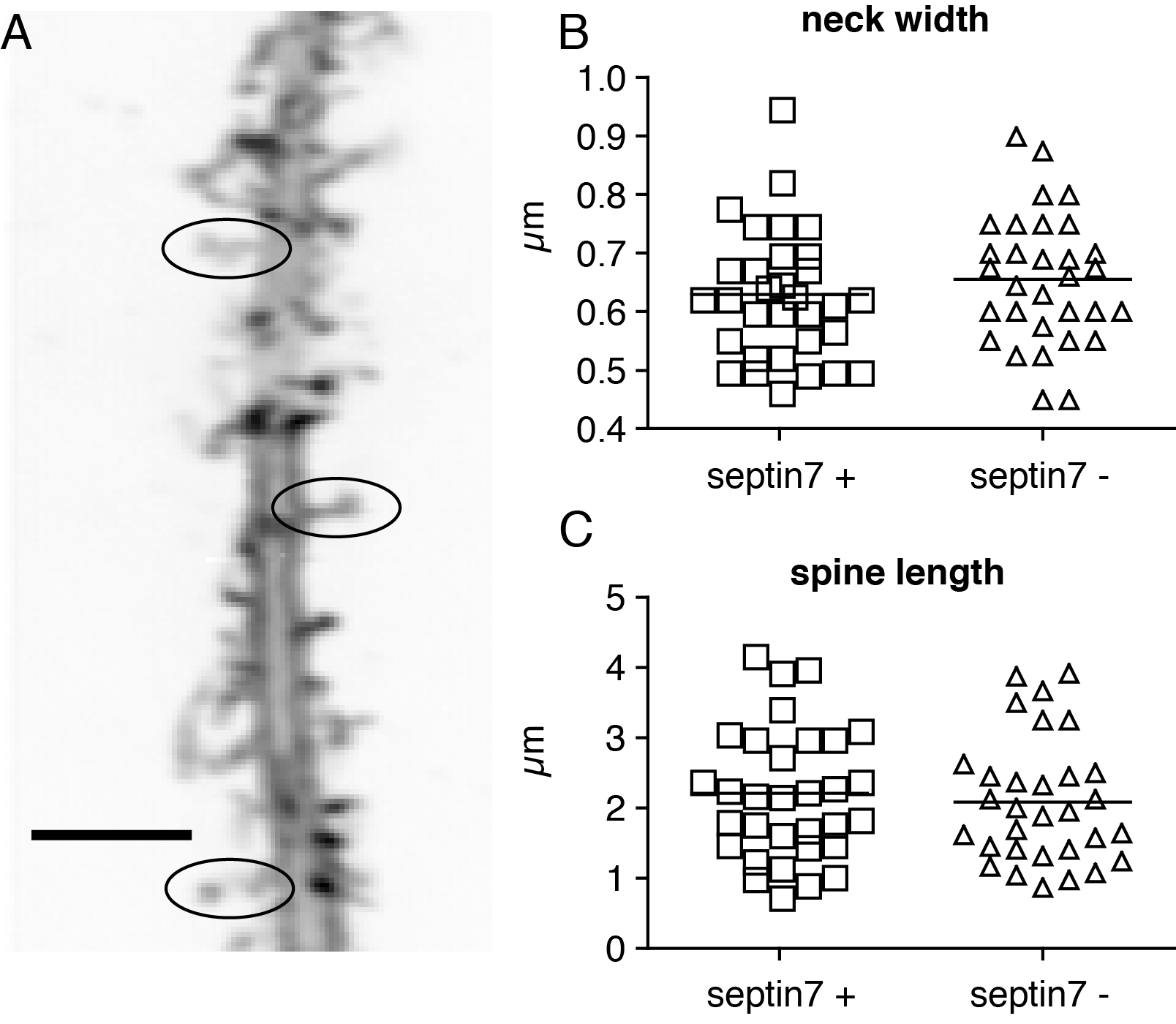

Supplement: S4 Figure — Shape of spines selected for FRAP experiments. To rule out the influence of spatial constraints on the outcome of FRAP experiments, spines of similar apparent shape were selected for experiments. (a) Inverted fluorescence image of CD4-mRFP fluorescence in a dendrite. Selected spines are marked. Scale bar is 5 µm. (b) The average apparent neck width in µm of selected spines in septin positive and septin negative spines as measured from full width at half maximum of spine neck fluorescence profile. p = 0.3088. (c) Average spine length in µm of selected spines in septin-positive vs. septin negative spines. p = 0.5919. Mann-Whitney test. (TIF) [file pone.0113916.s004.tif]
